# Public health laboratory systems development in East Africa through training in laboratory management and field epidemiology

**Published:** 2011-12-15

**Authors:** Fausta Mosha, Joseph Oundo, David Mukanga, Kariuki Njenga, Peter Nsubuga

**Affiliations:** 1 Ministry of Health and Social Welfare, Dar es Salaam, Tanzania; 2 Centers for Disease Control and Prevention, Nairobi, Kenya; 3 African Field Epidemiology Network, Kampala, Uganda; 4 Centers for Disease Control and Prevention, Atlanta, Georgia, USA

**Keywords:** FELTP, Tanzania, Kenya, laboratory epidemiologists

## Abstract

Laboratories are integral to the delivery of quality health care and for public health functions; however laboratory systems and services are often neglected in resource-poor settings such as the East African region. In order to sustainably strengthen national laboratory systems in resource-poor countries, there is a need to train laboratory personnel to work in clinical as well as public health laboratories. In 2004,Kenya, Uganda, Tanzania, and South Sudan began training public health laboratory workers jointly with field epidemiologists in the Kenya Field Epidemiology and Laboratory Training Program (FELTP), and later through the Tanzania FELTP, as a strategy to strengthen public health laboratories. These programs train laboratory epidemiologists through a two-year public health leadership development course, and also offer various types of short course training for frontline staff. The FELTP laboratory graduates in Kenya, Tanzania, Uganda, and South Sudan are working in their respective countries to strengthen public health laboratory systems while the short course participants provide a pool of frontline implementers with the capacity to support the lower tiers of health systems, as well as serve as surge capacity for the regions and the national level. Through training competent public health laboratory workers, the East African ministries of health, in collaboration with other regional partners and stakeholders are now engaged in developing and implementing a holistic approach that will guarantee an overall strengthening of the health system by using well-trained public health laboratory leaders to drive the process. Strengthening public health laboratory medicine in East Africa is critical to improve health-care systems. The experience with the FELTP model in East Africa is a step in the right direction towards ensuring a stronger role for the laboratory in public health.

## Background

Sub-Saharan Africa carries a massive burden of infectious and non-infectious diseases. However laboratories which would enable clinicians to make accurate diagnoses, offer correct treatment, and address epidemic prone diseases, are ill equipped and poorly resourced [1]. Although laboratories are integral to the delivery of quality health care and for public health functions, laboratory systems and services are often neglected in resource-poor settings such as the countries in East Africa [2]. East African laboratories are also disproportionately affected by staff shortages, poor communication facilities, inadequate equipment, low staff morale, and lack of training [1]. Public health laboratories have almost disappeared in the East African region as they have either been integrated with, or replaced by, clinical diagnostic laboratories [3]. Where public health laboratories exist, they are at the national level only and are characterized by shortages of essential staff and equipment.

In 1998, the World Health Organization (WHO) Regional Office for Africa (AFRO) proposed the Integrated Disease Surveillance and Response (IDSR) approach for improving public health surveillance and response in Africa through linking community, health facility, district, and national levels [4]. IDSR also promotes rational use of resources by integrating and streamlining common surveillance activities. Surveillance activities for different diseases involve similar functions and often use the same structures, processes and personnel [5].

In what has become the largest ever investment to control a specific disease, the United States (U.S.) government initiated the President's Emergency Plan for AIDS Relief (PEPFAR) in 2003. This plan was reauthorized through the Lantos-Hyde legislation in 2010 and is the cornerstone of the U.S. President's Global Health Initiative (GHI) [6,8]. PEPFAR aims to provide prevention, treatment, and care to turn the tide of the HIV epidemic that disproportionately affects poor sub-Saharan African countries. The success of PEPFAR and GHI are inextricably linked to the ability to have reliable testing and confirmation of a series of diseases and syndromes through reliable, effective public health and clinical laboratories which are led by well-trained nationals from the countries that are affected by HIV, tuberculosis, and malaria as part of strengthened public health systems that are owned and operated, and led by ministries of health.

In order to build capacity for public health surveillance in public health laboratories in East Africa, a major investment in personnel and equipment for national public health laboratory networks is needed. Additionally, broader knowledge, skills, and competencies in best practices are essential in implementing innovative strategies to improve the quality of laboratory testing in resource limited environments like the East African region. Since the adoption of IDSR strategy, there have been efforts to strengthen the overall national system for the surveillance of diseases including laboratory confirmation of suspected outbreaks in line with the requirements of the revised International Health Regulations. Sustainable progress can only happen if laboratories are represented by laboratory personnel on key decision-making bodies, rather than being represented by other sections of health care services, such as the pharmacy or radiology section which is the practice in the East African region [3].

In order to strengthen national laboratory systems in resource-poor countries sustainably, there is a need to develop national laboratory strategic plans and policies, establish public-private partnerships, and to ensure effective leadership, commitment and coordination by host governments. Centers of excellence in field epidemiology and public health laboratory practice also need to be developed, or strengthened where they currently exist, and the workforce will need to be developed to operate these new public health systems [4,9]. Studies have shown that education and training of laboratory personnel improves the quality of test results and clinicians’ trust in the laboratory and their subsequent willingness to remain in an under-developed area for a longer time [10,11].An efficient laboratory can dramatically reduce waiting time to get results and leads to faster and better health care delivery. One need in public health laboratories today is to develop workers to carry out new and highly complex procedures. These workers must also learn to use new automated testing equipment and master the theories behind the new tests involved while developing new the skills [13]. The Field Epidemiology and Laboratory Training Program (FELTP) is a 2-year, full-time postgraduate applied public health training program for public health leaders [14]. It is modeled after the 60-year-old U.S. Centers for Disease Control and Prevention (CDC) s Epidemic Intelligence Service, which trains field epidemiologists to operate public health surveillance and response systems in the U.S. and has been adapted internationally as the Field Epidemiology Training Program (FETP) [15]. The FELTP which enrolls laboratory scientists (in the laboratory epidemiology track) in addition to physicians and other health scientists (in the field epidemiology track), was developed as a tool to develop laboratory epidemiologists to operate public health laboratories and networks, and field epidemiologists to operate public health surveillance and response systems. The FELTP is adapted to suit local contexts [16].

FELTPs and FETP shave yielded long-term, sustained results by reinforcing a culture of evidence-based practice and providing cadres of competent, motivated public health professionals able to respond to a multitude of public health threats [15].The laboratory-specific goal of an FELTP is to establish functional laboratory-based disease surveillance systems for priority diseases with an enhanced laboratory capacity to guide outbreak response. The addition of a laboratory training component begins to address the long-standing disconnect between field epidemiology and laboratory practice. FELTPs are meant to build and strengthen public health laboratory networks. FELTP training emphasizes competencies in epidemic preparedness, outbreak investigation and response, emerging infectious disease surveillance, and pathogen diagnostic techniques which are integrated in the field epidemiology and public health laboratorians training program.

## Approach and results

The Kenya FELTP was established in 2004 as a collaborative partnership led by the Kenya Ministry of Health (MOH), including the Jomo Kenyatta University of Agriculture and Technology, and CDC with funding from the Ellison Medical Foundation. This program was also tasked to train laboratory epidemiologists from other parts of Africa, including Ghana, South Sudan, Tanzania, and Uganda [13].Currently, the program trains public health professionals from Kenya and South Sudan.

The Tanzania FELTP was established in 2008 by graduates of the Kenya FELTP. It is a collaborative partnership of multiple stakeholders led by the Ministry of Health and Social Welfare (MOHSW) including Muhimbili University of Health and Allied Sciences (MUHAS), CDC, the African Field Epidemiology Network (AFENET) and the National Institute for Medical Research (NIMR).

Both the Kenya and Tanzania FELTPs place emphasis on provision of service during training, thereby providing real-time results to the host country ministries of health. FELTP trainees in Kenya and Tanzania receive an award of Masters of Science degree upon successful completion of the training.

The Kenya and Tanzania FELTP shave limited classroom instruction (25%) with most of the time (75%) spent in field assignments. The laboratory residents undertake courses in field epidemiology, biostatistics, research methodology, scientific communication, public health surveillance, computers in public health, advanced laboratory methods, laboratory management, laboratory methods in the field, and management and leadership. Didactic training sessions are carried out through combinations of presentations, videos, practical exercises, seminars, open discussions, wet laboratory exercises, and university examinations. A thesis is also completed during the second year of residency.

For their field placements, laboratory residents are placed in national and regional or provincial laboratories under the supervision of the Laboratory Resident Advisors who are experienced doctoral level public health laboratory scientists, and mentorship of experienced laboratory managers. The Laboratory Resident Advisors take leadership in guiding, teaching, and supervising the residents throughout the 2-years along with the onsite laboratory supervisors. During the field assignment, the residents conduct evaluations of epidemiologic and laboratory-based surveillance systems, perform disease control and prevention measures including outbreak investigations and design laboratory quality improvement projects. They report their findings to decision and policy makers and also train other health workers.

Upon completion of the program, laboratory epidemiologists are trained to be able to: Routinely analyze the quality of laboratory data to identify possible aberrations and trends and recommend improvements; Design or evaluate a laboratory-based surveillance system, for example influenza, measles, and tuberculosis surveillance systems; Collaborate with field epidemiologists in an outbreak investigation or field–based study; Interpret and communicate laboratory results of public health importance to decision makers; Use management skills to create or support national and international public health laboratory networks; Use quality management systems to provide timely and accurate laboratory services; Integrate the core function and capabilities of the public health laboratory into existing clinical laboratories.

### Short courses

FELTPs also conduct short courses for existing laboratory personnel (and surveillance personnel) who are not able to participate in the 2-year program. The topics that are covered in the short courses include basic field epidemiology, biostatistics, disease surveillance, outbreak management, laboratory management, biosafety and biosecurity. The aim of the short courses is to create a pool of frontline implementers with capacity to support the functions of public health laboratories from the lower tiers of the health systems [16].The short courses are conducted in the form of a 2-week workshop followed by a 3-month applied learning project period, during which time trainees conduct a field project at their worksite. The types of projects that short course participants work on include outbreak investigations, laboratory quality improvements, and surveillance data analysis. After the field work, participants present the project findings to their facilitators, mentors, FELTP staff and residents, partners, and ministry of health policy makers during a 1-day graduation seminar. These short courses have increased the number of health workers that are trained using a competency-based approach to address public health problems. The short courses multiply the effect of the FELTP beyond the small numbers residents that are trained in the 2-year program, given each FELTP cohort are averages 13-15 residents per year, whereas each short course intake is about 30 participants and a FELTP is able to teach at least two short courses per year. Many of the short courses are taught by residents of the 2-year FELTP. Ultimately, the network of short course graduates and 2-year graduates will measurably improve the operation of public health surveillance and response systems in the East African region.

### Output to public health

The FELTP laboratory graduates have been working in their respective countries to strengthen public health laboratory systems though provision of: Timely laboratory confirmation of disease pathogens for surveillance, outbreak response and prevention. Most of the current suspected outbreaks are being investigated by FELTP laboratory graduates and the causative agents are identified in a timely way; Policy, standards, and advocacy for quality public and private laboratory services, through supporting the laboratories in the region towards accreditation; Training and continuing education for laboratory personnel on surveillance and outbreak investigation through short courses; Scientific and managerial leadership in development of public health policy, as evident by existing laboratory strategic plans; Research and development capacities; Writing and disseminating manuscripts and publications.

## Discussion

The recent focus by ministries of health in East Africa on strengthening health systems and the emphasis on laboratory systems suggest that the opportunity has presented itself for the international community to act now, act collectively, but act differently to ensure sustainability of global health efforts to enhance laboratory networks and systems [12]. The establishment and launching of the FELTPs, together with programs such as Strengthening Laboratory Management toward Accreditation (SLMTA) and the newly-established WHO AFRO laboratory quality improvement process towards accreditation, will provide an impetus to change the future of public health surveillance and response systems in East Africa to include strengthening of the public health laboratories [13].

SLMTA, launched in Kigali, Rwanda as a preparatory initiative to prepare laboratories towards accreditation [17], was developed to promote immediate and measurable improvement in laboratories of developing countries. FELTP graduates and residents participate in implementing laboratory quality improvement processes using the SLMTA approach and also in training other laboratory professionals on Laboratory Quality Systems (LQS). SLMTA, in combination with LQS training, supports laboratories by improving management and building preparedness for accreditation.

Ultimately a locally recruited, well-trained public health workforce comprising field epidemiologists, public health laboratory epidemiologists, and short course graduates who are able to operate multi-disease surveillance and response systems will provide the basis on which national public health institutions can be built to sustainably prevent and control priority disease conditions in East African countries.

FELTP has contributed to enhancing the public laboratory strengthening component in ministries of health of participating countries, which has led to improved diagnostic and monitoring services, as well as implementation of laboratory quality management systems and initiation of laboratory accreditation process that is taking place in East Africa.

Through FELTPs, East African countries have managed to obtain well trained public health laboratory leaders to drive the process of strengthening laboratory systems. These graduates have been retained in their home countries and the nearby regions to support the laboratory improvement process (table 1).

**Table 1:** 2006 to 2010 graduates of Kenya and Tanzania Field Epidemiology and Laboratory Training Program and their current positions

	Graduation year	Programme	Country of residence	Current position	Organisation
**1**	2006	KFELTP	Kenya	Program Officer-Comitato Collaborazione Medica	Ministry of Health Somalia
**2**	2006	KFELTP	Kenya	Head-Laboratory Information Management System; National Public Health Laboratory Services	Ministry of Public Health and Sanitation Kenya
**3**	2006	KFELTP	Kenya	Lab Resident Advisor	FELTP Rwanda
**4**	2007	KFELTP	Kenya	National Public Health Biochemistry Lab	Ministry of Public Health and Sanitation Kenya
**5**	2007	KFELTP	Uganda	Program Officer (Laboratory Quality Assurance) Central Public Health Lab	Ministry of Health Uganda
**6**	2008	KFELTP	Kenya	Field Coordinator	FELTP Kenya
**7**	2008	KFELTP	Kenya	Standards Officer	Ministry of Public Health and Sanitation Kenya
**8**	2008	KFELTP	Tanzania	Director, National Health Laboratory Services	Ministry of Health and Social Welfare Tanzania
**9**	2009	KFELTP	South Sudan	Deputy Team Leader,	MDTF Health project Eastern, Equatorial state, South Sudan
**10**	2009	KFELTP	Kenya	Assistant Research Officer	CDC Kenya
**11**	2009	KFELTP	Kenya	Laboratory Manager	Kenya Army
**12**	2009	KFELTP	Uganda	Medical Research (Laboratory) Specialist	CDC Uganda
**13**	2010	KFELTP	South Sudan	Program Manager/SPLA HIV secretariat	Government of South Sudan
**14**	2010	KFELTP	South Sudan	Senior Inspector of Public Health Laboratories	Ministry of Health Government of South Sudan
**15**	2010	KFELTP	Kenya	Laboratory Epidemiologist-Eastern Province	Ministry of Public Health and Sanitation Kenya
**16**	2010	KFELTP	South Sudan	Research Officer, Ministry of Animal Resource	Ministry of Health Government of South Sudan
**17**	2010	KFELTP	Kenya	Assistant Research Officer	Kenya Medical Research Institute/CDC
**18**	2010	KFELTP	Kenya	Assistant Lecturer Medical Laboratory Sciences	Mombasa Polytechnic University College
**19**	2010	TFELTP	Tanzania	Laboratory Epidemiologist	Defence Force Tanzania
**20**	2010	TFELTP	Tanzania	Laboratory Epidemiologist	Ministry of Health and Social Welfare Tanzania
**21**	2010	TFELTP	Tanzania	Laboratory Epidemiologist	Ministry of Health and Social Welfare Tanzania
**22**	2010	TFELTP	Tanzania	Laboratory Epidemiologist	Muhimbili University of Health and Allied Sciences
**23**	2011	KFELTP	Kenya	Laboratory epidemiologist	Ministry of Public Health and Sanitation

Knowledge and skills of laboratory personnel are fundamental for the implementation of a laboratory quality management system and FELTPs are critical to the improvement of laboratory management in East African countries. The progress of laboratories in East African countries will depend on the efforts made to identify opportunities for creating and streamlining resources to achieve functional national laboratory networks that meet national priorities and support global disease program objectives including training more laboratory epidemiologists. The FELTP laboratory graduates are beginning to build an East African public health laboratory network through their relationships and support from the African Field Epidemiology Network (AFENET). AFENET has a series of laboratory strengthening activities that it conducts with funding from a number of donors including CDC and the Bill and Melinda Gates Foundation.

## Limitations

The FELTP model is still a work in progress in East Africa. The first FELTPs are less than 10 years old and there are several lessons to learn, especially the usefulness of the competencies acquired during the training period. Uganda has yet to develop a fully-fledged FELTP and the results in South Sudan are evolving. Career paths of laboratory epidemiologists will need to be developed, and this depends on having a critical mass of graduates, which also requires sustained funding particularly from the region's ministries of health, which are facing several other challenges. However, the early lessons from the graduates of the public health laboratory component show that this model can be successful.

## Conclusions

Strengthening public health laboratory medicine in East Africa is critical to improve health-care systems. Apart from training of laboratory personnel, laboratory departments should be created within the ministries of health, in tandem with strong leadership and together with involving laboratories in planning stages. The experience with the FELTP model in East Africa is an early step in the right direction towards ensuring a stronger role of the laboratory in public health. Ministries of health in East Africa and the rest of sub-Saharan Africa should consider the FELTP model as one locally-adaptable tool to create the health workforce that can both strengthen field epidemiology and laboratory management in one program that is led and owned by them. Sustained funding of this effort and the development of appropriate career paths will need continued attention from host governments and their donor partners. Ultimately FELTPs can be the basis on which national public health institutions for disease prevention and control can be developed in resource constrained countries.
